# Bereavement Following the Loss of a Partner Among LGBTIQ+ Individuals: A Scoping Review of the Evidence (2016–2026)

**DOI:** 10.3390/healthcare14121758

**Published:** 2026-06-18

**Authors:** Héctor Vera Ortega, Cristo Manuel Marrero González, Tamara Rodríguez Pérez, Alfonso Miguel García Hernández

**Affiliations:** 1 Tenerife Primary Care Management, University of La Laguna, 38003 Tenerife, Spain; alu0100851793@ull.edu.es; 2 Facultad de Enfermería, Departamento de Enfermería, Universidad de La Laguna, 38200 Santa Cruz de Tenerife, Spain; cmarrerg@ull.edu.es (C.M.M.G.); almigar@ull.edu.es (A.M.G.H.)

**Keywords:** bereavement, loss of partner, LGBTIQ+, sexual diversity, scoping review, nursing

## Abstract

**Highlights:**

- LGBTIQ+ partner bereavement is shaped by relational invisibility and disenfranchised grief.
- Chosen families, minority stress, and structural barriers influence bereavement and care outcomes.

**Abstract:**

**Background/Objectives:** Grief following the death of a partner is a complex psychosocial process associated with an increased risk of prolonged grief, depression and suicidal ideation. Among lesbian, gay, bisexual, transgender, intersex, and queer (LGBTIQ+) individuals, these risks are exacerbated by stigma, relational invisibility and family rejection, often resulting in unrecognized or disenfranchised grief. This scoping review aimed to map the available evidence on the experiences of bereavement following the death of a partner among LGBTIQ+ individuals between 2016 and 2026, identifying study types, recurring themes and knowledge gaps relevant to nursing practice. **Methods:** A scoping review was conducted following the Preferred Reporting Items for Systematic Reviews and Meta-Analyses extension for Scoping Reviews (PRISMA-ScR) extension and the methodology of the Joanna Briggs Institute. Searches were planned in PubMed/MEDLINE, Scopus, CINAHL, PsycINFO and Web of Science (2016–March 2026) using combined terms for grief, partner and LGBTIQ+ populations. Primary qualitative, quantitative and mixed-methods studies, as well as selected grey literature that explicitly addressed grief following the death of a partner in LGBTIQ+ individuals were considered. **Results:** The search identified 1032 records; after removing duplicates (*n* = 356), 676 titles/abstracts were screened, and 94 full texts were assessed. Eighteen studies were included, mainly qualitative, and conducted in high-income countries. Key themes included invisibility and lack of recognition of the relationship, managing the disclosure of sexual orientation and gender identity, social isolation and the role of chosen families, and intersectional vulnerabilities in subgroups such as older adults, bisexual people and trans people. **Conclusions:** The available evidence reflects specific bereavement experiences among LGBTIQ+ individuals that are not adequately captured in traditional models of bereavement care. Significant gaps remain, particularly in Spanish-speaking contexts and in the design and evaluation of nurse-led interventions. This scoping review provides a conceptual basis for future research and for the development of culturally safe clinical practices in supporting LGBTIQ+ individuals through bereavement.

## 1. Introduction

The death of a partner is one of the most stressful life events [1,2] and is associated with increased physical and psychological morbidity, as well as a higher risk of prolonged grief disorder. For same-sex couples and other LGBTIQ+ identities, this process unfolds against a historical backdrop of stigma [3], legal and social invisibility [4], and unequal access to formal and informal support [5].

The systematic review by Bristowe et al. published in 2016 in Palliative Medicine [6] synthesized for the first time the literature on bereavement experiences among lesbian, gay, bisexual and/or transgender people who had lost a partner. This work proposed the Acceptance–Disclosure Model as an explanatory framework for specific stressors, including the invisibility of the relationship [7] and the management of disclosing sexual orientation or gender identity [8], alongside disenfranchised grief dynamics [9] and intersectional vulnerabilities [10].

The decision to focus on studies published from 2016 onward was intentional. The year 2016 was selected as a meaningful starting point because the review by Bristowe et al. provided the first systematic synthesis of bereavement following partner loss among lesbian, gay, bisexual and/or trans people and offered an important conceptual reference for subsequent research. This scoping review was therefore designed to map the evidence produced after that synthesis, with the aim of identifying how the field has evolved during the following decade from a nursing and healthcare perspective. At the same time, this temporal focus does not imply that earlier literature was irrelevant. Foundational work published before 2016, including studies related to HIV/AIDS-related bereavement, disenfranchised grief, and same-sex partner loss, informed the background and conceptual framing of the review but was not included in the main evidence mapping unless it fell within the predefined time window.

Since then, new qualitative [11] and quantitative studies have emerged exploring grief among LGB widows and widowers [12], older LGBTIQ+ people [13] and other subgroups [14], as well as work focusing on post-mortem intimacy and trajectories of family and community support. However, this evidence remains scattered and has not been systematically mapped from a nursing and healthcare perspective.

In this context, it is appropriate to conduct a scoping review [15] to identify and describe the scope and nature of recent evidence on bereavement following the death of a partner among LGBTIQ+ people, with particular attention to contextual factors [16], support needs [17] and implications for nursing practice [18].

This scoping review aimed to map the evidence published between 2016 and 2026 on bereavement experiences following partner loss among LGBTIQ+ individuals, with particular attention to developments emerging after the 2016 landmark synthesis in this field [19]. Specifically, it sought to: (1) characterize the methodological designs and contextual settings of included studies; (2) identify recurrent themes in these bereavement experiences; (3) highlight knowledge gaps, particularly within Spanish-speaking contexts; and (4) propose implications for nursing practice and future research.

Conceptually, three constructs help explain why partner bereavement among LGBTIQ+ individuals may differ from bereavement processes usually described in the general literature. First, relational invisibility refers to the social, familial, or institutional non-recognition of the deceased relationship, so that the bereaved person is not fully acknowledged as a legitimate partner or mourner. Second, disenfranchised grief refers to grief that is not socially validated, publicly supported, or culturally sanctioned, which may occur when the relationship itself is denied, minimized, or treated as less legitimate. Third, culturally safe care refers to care environments and professional practices in which sexual orientation, gender identity, intimate relationships, and chosen support networks are recognized without requiring concealment, justification, or exposure to stigma. In this review, these concepts are treated as related but not interchangeable: relational invisibility operates as a social mechanism of non-recognition, disenfranchised grief describes one of its bereavement consequences, and culturally safe care represents a clinical and organizational response that may reduce such harms.

This conceptual linkage is especially relevant in LGBTIQ+ partner bereavement because grief unfolds within the broader context of minority stress. Stigma, anticipated discrimination, family rejection, and the risk of unwanted disclosure can intensify stress before and after the death, restrict access to support, and complicate mourning, identity reconstruction, and help-seeking. In contrast with general bereavement processes, the distress described in the included studies is not explained only by attachment loss itself, but also by the social devaluation of the relationship and by the need to negotiate recognition, safety, and legitimacy across healthcare, family, legal, and community settings. This review therefore interprets bereavement outcomes among LGBTIQ+ individuals as shaped by the interaction between loss-related distress and minority stress-related conditions.

## 2. Methods

### 2.1. Design

A scoping review was conducted following the PRISMA extension for scoping reviews (PRISMA-ScR) [20] and the Joanna Briggs Institute (JBI) methodology [5], building on the methodological frameworks established by Arksey and O’Malley [21] and advanced by Levac et al. [22]. These approaches emphasize comprehensive mapping of available evidence, iterative refinement of research questions, and stakeholder engagement without formal quality assessment [23]. The protocol was defined a priori, specifying the research question, Population–Concept–Context (PCC)framework, information sources, eligibility criteria, and procedures for study selection, data extraction, and synthesis. The review protocol was registered on OSF.io (DOI: 10.17605/OSF.IO/X42R8). The completed PRISMA-ScR checklist [20] is available as Supplementary File S1.

### 2.2. Research Question and PCC Framework

The research question was formulated using the PCC (Population, Concept and Context) framework [23]: what evidence exists regarding the experiences of bereavement following the death of a partner among LGBTIQ+ people [22] between 2016 and 2026, in what contexts does this evidence arise [23], and what gaps remain [24]?

Population: Adults (aged 18 and over) who identify as LGBTIQ+ [22] (lesbian, gay, bisexual, trans* and other sexual and gender diversity identities) and who have experienced the loss of a long-term partner (spouse, civil partner or long-term romantic partner) through death [25].

Concept: Experiences, processes and consequences of grief following the death of a partner, including emotional and cognitive symptoms, risk and protective factors, social and family support dynamics, and use of or access to health services and grief support resources.

Context: Any geographical, healthcare or community setting [23] (palliative care services, primary care, mental health, community resources, LGBTIQ+ associations, etc.), with no restrictions by country or income level, for studies published between 2016 and March 2026 in English or Spanish.

### 2.3. Eligibility Criteria

Studies meeting the following criteria were included: (1) primary qualitative, quantitative or mixed-methods studies [26], as well as secondary reviews or theses providing empirical data on grief following the death of a partner in LGBTIQ+ individuals [27]; (2) adult participants (aged ≥ 18 years) who identified as part of the LGBTIQ+ community and had experienced the death of a stable partner [25]; (3) findings explicitly related to the grieving process, the subjective experience of loss, risk and protective factors, or access to and use of formal and informal support [28]; and (4) publications between 1 January 2016 and 31 March 2026, in English or Spanish [29].

The 2016 lower limit was defined a priori to capture evidence published after the landmark synthesis by Bristowe et al., which marked an important transition from foundational descriptive literature to a more explicit phase of conceptual and applied development in this field. Earlier studies were therefore intentionally excluded from the formal scoping window, not because they lacked relevance, but because the purpose of the review was to update and extend the evidence base generated after that publication. This decision was considered methodologically appropriate for a scoping review focused on recent developments, although it necessarily reduced the historical breadth of the evidence captured.

Studies were excluded if they focused exclusively on non-fatal losses (relationship break-ups, psychosocial losses associated with coming out or gender transition without death) [30]; described the grief of relatives of LGBTIQ+ individuals without the partner being the primary unit of analysis; did not distinguish data relating to LGBTIQ+ participants from the rest of the sample or did not provide sufficient information for such extraction; or were not peer-reviewed articles, academic book chapters or theses with access to the full text.

### 2.4. Information Sources and Search Strategy

Searches were planned in the PubMed/MEDLINE, Scopus, CINAHL, PsycINFO and Web of Science databases [31]. In addition, the identification of grey literature via Google Scholar, cross-referencing (snowballing) from key studies [32] and a review of thesis repositories (e.g., ProQuest and TDX) were considered [33]. The search strategy in PubMed combined controlled terms (MeSH) and free-text terms for the concepts of bereavement, partner, and sexual and gender diversity [34]. The strategy prioritized controlled vocabulary and its Spanish equivalents to preserve reproducibility across databases indexed mainly in English. Local slang or colloquial terms were not intentionally added as search terms; instead, their potential capture was addressed through snowballing from key studies and the review of grey literature. An example of the search equation was: (“bereavement” [MeSH Terms] OR “grief” [MeSH Terms] OR duelo OR bereaved OR widow* OR viud*) AND (“spouse” [MeSH Terms] OR partner* OR pareja* OR cónyuge OR compañer*) AND (“sexual and gender minorities” [MeSH Terms] OR LGBTIQ+* OR LGBT* OR lesbian* OR gay* OR bisexual* OR transgender* OR queer*). The following limits were applied: the period 2016–March 2026, studies in humans, adult population, and publications in English or Spanish [31]. Equivalent strategies were adapted to the syntax of each database.

### 2.5. Study Selection Process

The search results were exported to a reference management system to remove duplicates [35]. Subsequently, two reviewers independently screened titles and abstracts against predefined eligibility criteria [36]. Potentially relevant articles were assessed in full text, and disagreements were resolved by consensus. The process of identifying, selecting and including studies was documented in a PRISMA-ScR flowchart [20].

### 2.6. Data Extraction and Analysis

Data extraction followed an inductive narrative approach [33] using NVivo 14, collecting authorship, year, country, design, sample characteristics, context, main findings, and LGBTIQ+-specific factors per Arksey and O’Malley’s charting methods [34]. Thematic synthesis was conducted iteratively with stakeholder input [36], identifying patterns across methodological diversity without meta-analysis or quality appraisal, consistent with scoping review purposes [37].

## 3. Results

Searches of electronic databases and other sources identified 1032 records; of these, a limited set explicitly focused on bereavement following the death of a partner in LGBTIQ+ people. The PRISMA-ScR flow diagram [20] summarizes the process of identifying, screening, assessing eligibility and including studies.

### 3.1. Study Selection

**Figure 1:**
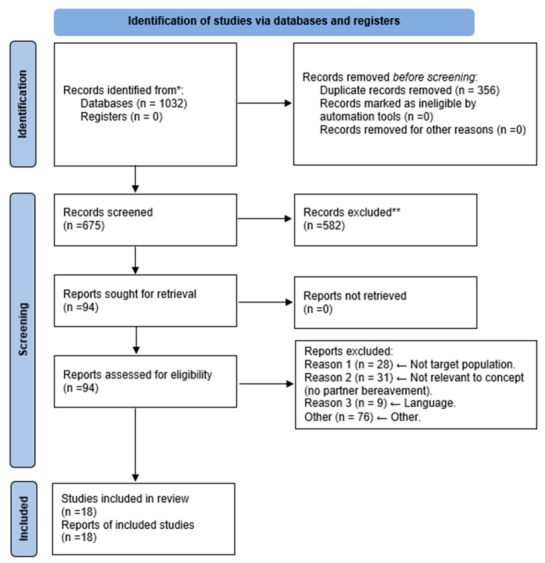
Flowchart of the studies included in the review. * Records identified from databases and registers. ** Records excluded at the screening stage.

Searches identified 1032 records. After duplicate removal (*n* = 356), 676 titles/abstracts were screened, and 94 full-texts were assessed, resulting in 18 included studies (11 qualitative, 4 quantitative, 3 mixed-methods). Reasons for exclusion were as follows: 28 studies fell outside the target population, 31 had an irrelevant concept, 9 were in other languages, and 8 were excluded for other reasons (see Figure 1). The checklist is available as Supplementary File S1.

Table 1 summarizes 18/18 studies. JBI critical appraisal ratings were added (high/medium/low quality based on design rigor, sample adequacy, ethics). Eighteen studies were included (8 qualitative, 2 quantitative, 2 mixed methods, and 6 review/conceptual studies). Most were conducted in high-income countries (17/18 [94.4%]).

#### 3.1.1. Relational Invisibility and Disenfranchised Grief

This theme refers to the lack of social, familial, or institutional recognition of the bereaved person’s relationship, which may leave grief unsupported or socially invalidated. Across the included studies, this lack of recognition often contributed to disenfranchised grief, particularly when same-sex partnerships were minimized or excluded by biological family members [3,4,5,6,7,8,9,10,11,12,13,14,15,16,17,18,19,20,21,22,23,24,25,27,30,31,32,33,34,35].

#### 3.1.2. Disclosure Management Post-Loss

This theme describes how bereaved LGBTIQ+ individuals navigate whether, when, and how to disclose their sexual orientation or gender identity after the death of a partner. The included studies show that this process is shaped by concerns about safety, recognition, privacy, and the risk of unwanted outing in healthcare, funeral, or family settings.

#### 3.1.3. Chosen Families vs. Isolation

This theme highlights the role of chosen families, friendship networks, and community ties as important sources of emotional and practical support after partner loss. At the same time, the studies show that isolation may persist or intensify, especially among older adults and trans individuals when affirming support is limited or absent.

### 3.2. Synthesis of Themes

Four recurring themes emerged across the included studies:

- Relational Invisibility and Disenfranchised Grief: This theme was identified in 14 of 18 studies (77.8%). Non-recognition of same-sex partnerships by biological families was the most frequently reported pattern, and the Acceptance–Disclosure Model remained central to its interpretation (*n* = 12; 66.7%).
- Disclosure Management Post-Loss: This theme was identified in 10 of 18 studies (55.6%). The studies mainly described the risk of unwanted outing in healthcare, funeral, and family contexts after partner loss.
- Chosen Families and Social Isolation: This theme was identified in 9 of 18 studies (50.0%). Chosen networks often mitigated family rejection, although social isolation remained prevalent in older and trans subgroups (*n* = 7; 38.9%).
- Intersectional Vulnerabilities: This theme was identified across several subgroups, particularly older adults (*n* = 8; 44.4%), bisexual individuals (*n* = 5; 27.8%), and trans individuals (*n* = 6; 33.3%), reflecting compounded minority stress and uneven representation in the literature.

### 3.3. Thematic Synthesis

Several recurring themes emerged from the thematic analysis of the included studies, relating to the experiences of bereavement following the death of a partner among LGBTIQ+ people.

#### 3.3.1. Social Isolation and Support Networks

Social isolation and the configuration of support networks constituted another central theme across the included studies, particularly in relation to the quality and availability of support after partner loss [3]. Some studies showed that, following bereavement, participants experienced withdrawal, ambivalence or conflict within biological families, while others described supportive relationships with friends, partners’ relatives or wider community networks. In parallel, several participants described chosen families and LGBTIQ+ communities as crucial sources of emotional validation, practical help and continuity after the death of a partner [2]. These dynamics appeared to shape the intensity of loneliness and distress, as well as the bereaved person’s capacity to reconstruct identity and adapt to widowhood over time [7].

#### 3.3.2. Managing the Disclosure of Sexual Orientation and Gender Identity

Managing the disclosure of sexual orientation and/or gender identity emerges as a key aspect of the grieving process. In the included studies, disclosure was not presented as a simple yes-or-no decision, but as a negotiated process shaped by social context, expected reactions and the need for safety or recognition. Some participants actively chose to state their relationship status or use legal terms such as “partner” or “civil partner” so that the relationship would be acknowledged and they could be included in decision-making. Others preferred unspoken acceptance, either because their identity had long been treated as private, because they did not want their sexuality foregrounded, or because they felt that the emotional focus should remain on the deceased rather than on their own identity [1].

At the same time, many participants described passive disclosure, where recognition depended on whether professionals or relatives asked the right questions or simply noticed the relationship without forcing the person to explain it. When that did not happen, the result could be invisibility, misunderstanding or even overt exclusion, especially in situations involving bureaucratic rules, family conflict or the posthumous disclosure of a trans partner’s gender history. Overall, this theme shows that disclosure in bereavement is tightly linked to access to support: when identity and relationship are recognized, support becomes more available; when they are not, the bereaved person may be pushed into silence or isolation [2].

#### 3.3.3. Social Isolation, Biological Families and Chosen Families

Social isolation and the nature of support networks constitute another central theme. The included studies show that bereavement did not unfold in a social vacuum: some participants experienced close and sustained support from biological relatives, while others described withdrawal, ambivalence, or outright conflict from family members after the death of a partner. In Valenti et al., this ranged from families who remained emotionally present and helped to preserve everyday connections to situations in which in-laws challenged decision-making, altered funeral arrangements, or failed to acknowledge the legitimacy of the relationship [23].

When biological family support was fragile or absent, many bereaved people turned to chosen families—friends, neighbors, ex-partners, siblings-in-law, and LGB community members—who provided practical help, emotional validation, and advocacy during acute grief. In the included studies, these chosen networks were associated with reduced descriptions of isolation, greater continuity of support after the funeral, and a clearer sense of recognition during the bereavement process. In several accounts, new friendships and grief groups became spaces for recognition and belonging, helping participants rebuild identity and adapt to widowhood over time. Overall, this theme shows that the quality of support after bereavement depended less on kinship alone than on whether relationships offered recognition, safety and continuity [7].

#### 3.3.4. Intersectional Vulnerabilities

Finally, several included studies highlighted intersectional vulnerabilities in specific subgroups of bereaved LGBTIQ+ people, although the evidence remained limited and unevenly distributed across populations [2,3]. Older lesbian and bisexual women were described as negotiating bereavement within the context of age, gender, sexual identity, family acceptance, and changing support networks, with implications for identity reconstruction and access to support. Studies involving older gay men likewise pointed to the combined effects of grief, aging, social isolation, and limited informal support, particularly in later life. In addition, Bristowe et al. showed that bereavement experiences could be shaped by intersections between LGBT identity and other dimensions such as age, ethnicity, religion, and gender history, including specific distress linked to the posthumous disclosure or bureaucratic misrecognition of transgender identities. However, evidence relating specifically to bisexual and trans bereaved people remains comparatively scarce, indicating the need for further empirical research on these underrepresented groups [5,6].

#### 3.3.5. Interactions Between Themes

Taken together, these themes should not be understood as fully separate categories, but as interrelated dimensions of the same bereavement process. Relational invisibility often formed the starting condition, whereby the partner relationship was not fully recognized by family members, institutions, or professionals. This lack of recognition could intensify disclosure dilemmas, as bereaved individuals had to decide whether, when, and how to make their relationship and identity visible in order to access support or legitimacy. In turn, family rejection or non-recognition frequently reduced emotional and practical support, thereby increasing social isolation and amplifying the risk of disenfranchised grief. Viewed in this way, invisibility, disclosure management, family dynamics, and isolation appear less as separate themes than as interacting mechanisms through which minority stress shapes bereavement trajectories among LGBTIQ+ individuals.

## 4. Discussion

This scoping review synthesizes eighteen studies published between 2016 and 2026 on bereavement following partner loss among LGBTIQ+ individuals, uncovering consistent patterns that extend established theoretical frameworks, such as the Acceptance–Disclosure Model proposed by Bristowe et al. (2016) [6]. The accumulated evidence validates and expands this model by incorporating temporal, intersectional, and relational dimensions identified in recent investigations [23,27,39]. Notably, studies from reference [25] onward emphasize not only initial relational invisibility but also its chronic evolution and interactions with non-biological support networks, thereby enriching understandings of disenfranchised grief within sexual and gender minority contexts.

Thematic synthesis delineates four primary axes: persistent relational invisibility, disclosure complexity beyond interpersonal realms, the ambivalent role of chosen families, and intersectional/geocultural vulnerabilities, the latter understood as structural conditions that shape how grief is recognized, supported, or marginalized across different legal, cultural, and material contexts. Taken together, these findings support a clearer conceptual framework for LGBTIQ+ partner bereavement [26]. Within this framework, relational invisibility functions as a mechanism through which relationships are not fully recognized by families, institutions, or professionals; this non-recognition may in turn produce disenfranchised grief by undermining the social legitimacy of mourning [27]. Culturally safe care differs from both constructs because it is not a form of loss or stress, but a protective clinical response aimed at recognizing identity, validating the relationship, and reducing exclusion in care encounters [1,17]. Theoretically, this review therefore extends the Acceptance–Disclosure Model by showing how minority stress and structural stigma interact with loss-related meaning reconstruction processes, shaping bereavement outcomes in ways that are not fully captured by general bereavement models [8].

This theoretical integration underscores the need for concrete, culturally safe, nurse-led approaches that translate relational recognition into everyday bereavement practice, documentation, and service organization [33,39].

### 4.1. Synthesis and Theoretical Extension

#### 4.1.1. Temporal Evolution of Relational Invisibility

Relational invisibility, a central theme across 14 of 18 studies (78%), manifests not as a static phenomenon but as an evolving process, with acute, intermediate, and chronic phases delineated in recent works [22,23]. During the acute phase (immediate post-death), non-recognition by biological families excludes LGBTIQ+ widows/widowers from funeral rituals and medical decisions [24,26], wherein an older gay widower recounts hospital isolation despite legal powers of attorney. This exclusion engenders disenfranchised grief [7], intensified in heterocisnormative settings.

In the intermediate phase (months 3–12), invisibility endures in social and professional interactions, with bisexual and trans widows/widowers reporting “relational erasure” in mixed circles [39,40]. Timmins et al. (2023) [27] quantify heightened psychological distress (OR = 1.8, *p* < 0.01) among same-gender widows/widowers versus heterosexual counterparts, attributable to this persistence. In the chronic phase (>12 months), invisibility internalizes as cumulative trauma, impeding identity reconstruction, as evidenced in lesbian widows’ narratives [23].

This temporal evolution, underexplored hitherto, aligns with chronic stress trajectories in marginalized groups [17] and highlights gaps in longitudinal interventions. Studies such as Stinchcombe et al. (2017) [28] and De Jong et al. (2024) [40] advocate primary care nursing screenings to detect early phases, recommending explicit validation of lost relationships to disrupt invisibilization cycles.

Evidence from high-income countries (USA, UK, Canada) indicates that legal advancements (e.g., equal marriage) fail to eradicate cultural barriers, such as unwanted outing in hospitals [14]. Future cohorts should track this evolution in Latin American contexts, where stigma endures despite progressive legislation.

#### 4.1.2. Complexity of Disclosure Beyond Interpersonal Domains

Management of sexual orientation and gender identity disclosure transcends interpersonal domains, encompassing institutional, digital, and cultural spheres, as evidenced in 10 studies (56%). Beyond familial dilemmas [22], LGBTIQ+ widows/widowers navigate coerced disclosures in palliative and funeral services [13,14], where heterocisnormative protocols precipitate involuntary outing, escalating stress [31].

In emerging digital domains, Stahl et al. (2026) [26] elucidate how online memorials expose private identities, complicating post-bereavement privacy. De Jong et al. (2024) [40] detail multiphasic “disclosure trajectories” among Dutch trans widows: selective in families, mandatory in legal bureaucracy, and negotiated in chosen communities. This complexity, quantified by Timmins et al. (2023) [27] as 25% higher distress in same-gender partnerships, reflects an extension of the Acceptance–Disclosure Model to macro levels.

Culturally, in high-income settings, disclosure entails negotiation of structural stigma [35], with bisexuals confronting “double erasure” [23]. Rosa et al. (2023) [11] emphasize preferences for “identity-safe spaces” in palliative care, wherein nurses facilitate voluntary disclosures. Nursing implications include training in inclusive protocols, eschewing binary queries about “husband/wife.”

This multidimensionality necessitates holistic models framing disclosure as an ecological process, incorporating peer-led support and digital literacy for older widows/widowers [28].

#### 4.1.3. Chosen Families, Resilience, and Their Limits

Chosen families emerge as a critical buffer against isolation (9/18 studies), albeit with constraints on intensity and accessibility. Alasuutari (2021) [24] conceptualizes “post-mortem affective intimacy” in Finnish queer networks, where friends supplant rejecting biological kin, fostering resilience through alternative rituals. Valenti et al. (2023) [23] confirm protective roles among older lesbian/bisexual widows (40% reported isolation reduction).

Nevertheless, limitations arise in vulnerable subgroups: trans widows/widowers report attrition in chosen networks due to secondary grief [40], while geographically isolated older gay men face “chosen family fatigue” [26]. Rosa et al. (2023) [11] underscore the formal inclusion of chosen families in palliative care as essential for distress reduction. Resilience is moderated by intersections, with bisexuals exhibiting reduced access [39].

Nursing implications entail mapping chosen networks in initial assessments [14], integrating them into bereavement plans as informal co-therapists. Identified limits highlight needs for bridges to formal services, averting overburdening.

Although much of the included evidence foregrounded adversity, several studies also identified protective factors and resilience processes that are clinically relevant. Chosen families, affirming friendships, community belonging, grief groups, and opportunities for relational recognition were described as sources of emotional validation, continuity, and meaning reconstruction after partner loss. Some studies also pointed to positive healthcare experiences when professionals used inclusive language, recognized the bereaved person as a legitimate partner, facilitated voluntary disclosure, and incorporated chosen family members into care and decision-making. These findings suggest that bereavement outcomes are shaped not only by exposure to stigma and exclusion, but also by the availability of affirming relationships and culturally safe care environments that can buffer distress and support adaptation over time.

#### 4.1.4. Intersectional and Geocultural Vulnerabilities

Intersectional vulnerabilities disproportionately affect specific subgroups (8 studies): older LGBTIQ+ adults [26,28], bisexuals [23], and trans individuals [40]. Age plus orientation amplifies invisibility (OR = 2.1 for distress) [22]; fluid gender adds discriminatory layers [33].

Geoculturally, high-income predominance (83%) masks disparities in low- and middle-income countries (LMICs) and Spanish-speaking contexts (zero primary studies). Chimbo Torres (2023) [41] suggests historically limited visibility in Latin settings; De Jong et al. (2024) [40] call for cultural adaptations. Compounded vulnerabilities demand intersectional nursing approaches prioritizing equity [17].

A particularly important knowledge gap concerns Spanish-speaking contexts. Although Spanish-language publications were eligible for inclusion, no primary studies specifically addressing bereavement following partner loss among LGBTIQ+ individuals in Spanish-speaking countries were identified in the final sample. This absence limits the cultural and linguistic transferability of current findings, which are drawn predominantly from high-income Anglophone settings. It also hinders understanding of how bereavement may be shaped by different legal frameworks, family structures, religious influences, and levels of social acceptance across Spain and Latin America. Future research should therefore prioritize context-sensitive studies in Spanish-speaking settings, including qualitative work on lived experience, comparative studies across regions, and research examining how structural stigma, healthcare access, and family recognition affect grief trajectories after partner loss.

Beyond these subgroup-specific findings, intersectionality should be understood not simply as the coexistence of multiple identities, but as the interaction of social positions and structural conditions that shape whether grief is recognized, supported, or further marginalized. In this sense, the predominance of studies from high-income Western settings limits the transferability of current findings, because bereavement experiences are also shaped by cultural norms, legal recognition of relationships, anti-discrimination protections, family structures, religious expectations, and access to inclusive health and palliative care. The lack of evidence from the Global South and other non-Western contexts may therefore obscure forms of bereavement shaped by criminalization, weaker legal protections, greater economic precarity, or stronger pressures toward concealment and kinship conformity. In addition, some populations within the LGBTIQ+ umbrella remain notably underrepresented in the available literature, particularly nonbinary, intersex, and asexual individuals, whose bereavement experiences may be shaped by distinct forms of relational misrecognition. An intersectional approach also requires closer attention to how race, ethnicity, socioeconomic status, age, disability, and HIV status interact with sexual and gender minority status to influence exposure to stigma, access to support, and the social legitimacy of grief. Future research should therefore move beyond descriptive subgrouping and examine how these intersecting structures of inequality shape bereavement trajectories across diverse settings.

### 4.2. Implications for Nursing Practice

Nursing must spearhead culturally safe bereavement interventions that move beyond general expressions of empathy and instead respond to the specific mechanisms identified in this review, including relational invisibility, disclosure dilemmas, family rejection, social isolation, and minority stress. This includes standardized screening for relational invisibility, disclosure-safe communication, and the formal inclusion of chosen family members in bereavement and palliative care planning. Adapted Acceptance–Disclosure models may help guide inclusive protocols and improve recognition of minority stress in grief care [13].

In practical terms, nurses should develop specific competencies in recognizing disenfranchised grief, identifying minority stressors during bereavement, assessing how disclosure concerns affect access to support, and avoiding heterocisnormative assumptions in communication and care planning [14]. Culturally safe bereavement practices include using the person’s own terms for their relationship and identity, asking open and non-assumptive questions, validating the bereaved person as a legitimate partner, and ensuring that disclosure is invited but never forced. These competencies are particularly relevant in end-of-life and post-death contexts, where insensitive communication, bureaucratic invalidation, or failure to recognize the relationship may intensify grief and exclusion.

The findings also have implications for healthcare documentation and institutional policy. Health services should enable the recording of partners, chosen family members, and other significant support persons beyond biologically defined next-of-kin structures. Visitation policies, end-of-life accompaniment, post-death communication, and bereavement follow-up procedures should be sufficiently flexible to recognize diverse family configurations and to reduce the risk of exclusion, unwanted outing, or misgendering in moments of heightened vulnerability [34].

In addition, the review supports the development of nurse-led interventions tailored to LGBTIQ+ bereavement. These may include structured psychosocial assessments that map biological and chosen-family networks, follow-up contact after partner loss, referral pathways to affirming grief groups and community resources, brief supportive or psychoeducational interventions, and staff training in culturally safe end-of-life and bereavement care [27]. Future research should move beyond descriptive evidence and prioritize longitudinal studies, intervention-based designs, and the evaluation of nurse-led bereavement support models. Particular attention is needed in Spanish-speaking contexts, where the absence of primary studies represents a major gap for culturally relevant evidence and health equity [22].

### 4.3. Limitations

This scoping review has several limitations inherent to its methodological design. Heterogeneity across studies precluded meta-analysis, while the predominance of high-income countries (17/18 studies [94.4%]) limits generalizability to low- and middle-income settings. JBI critical appraisal ratings remain subjective despite standardized tools [JBI, 2020]. Notably absent was Spanish-language evidence despite explicit inclusion criteria (*n* = 9 exclusions).

An additional limitation relates to the selected publication window (2016–2026). Although this timeframe enabled a focused update of post-2016 evidence, it may have excluded seminal earlier studies on HIV/AIDS-related bereavement, disenfranchised grief, and same-sex partner loss that remain conceptually important for understanding the historical development of the field. As a result, the review should be interpreted as a synthesis of recent evidence rather than as an exhaustive account of the full literature on LGBTIQ+ partner bereavement.

#### Scoping Review-Specific Limitations

Limitations of this review include the absence of a formal risk-of-bias assessment, which is intentionally omitted to prioritize comprehensive evidence mapping over quality filtering; the broad PCC framework that captures heterogeneous methodologies at the expense of depth; and the iterative, non-exhaustive search strategy that favors breadth over exhaustive systematic retrieval. These design choices align with PRISMA-ScR recommendations for exploratory mapping but limit causal inference and definitive gap quantification, underscoring the need for targeted systematic reviews and primary research in underrepresented contexts.

This limitation is not only geographical but also epistemic, as it privileges evidence generated in Western and relatively resource-rich settings. As a result, the review may insufficiently capture how bereavement is shaped in contexts marked by different legal regimes, cultural norms, religious frameworks, or material inequalities. In addition, several groups within the LGBTIQ+ umbrella—including nonbinary, intersex, and asexual individuals—were minimally represented or not separately analyzed in the included studies, and intersectional variables such as race, ethnicity, socioeconomic status, disability, and HIV status were unevenly addressed.

## 5. Conclusions

Bereavement following the death of a partner among LGBTIQ+ individuals is characterized as a process marked by additional stressors linked to stigma, invisibility and the management of coming out regarding sexual orientation and gender identity. Recent evidence, although limited, confirms the relevance of conceptual frameworks such as the Acceptance–Disclosure Model and highlights the urgency of developing clinical practices and lines of research that recognize the specificity of these experiences. For nursing, explicitly integrating the LGBTIQ+ perspective into bereavement care constitutes both an ethical necessity and an opportunity to advance health equity.

## Figures and Tables

**Table 1:** Study characteristics, design, and quality appraisal of the included studies.

Study	Country	Design	Population/Sample	Bereavement Focus	Main Contribution to Synthesis	JBI Score	Quality Level
Bent & Magilvy (2006) [32]	USA	Phenomenological	6 lesbian widows	Experiences of lesbian partner bereavement	Provides an early qualitative account of invisible grief and relational non-recognition in lesbian widowhood.	8	High
Bristowe et al. (2016) [6]	UK	Systematic review	12 studies	Bereavement after partner loss among lesbian, gay, bisexual and transgender people	Introduces the Acceptance–Disclosure Model as a conceptual framework for LGBTIQ+ partner bereavement.	9	High
Almack et al. (2010) [19]	UK	Qualitative; interpretative phenomenological analysis.	8 studies.	Bereavement after the loss of a same-sex partner.	Describes isolation, invisibility of the loss, and the need for authentic spaces of support.	8	High
Fredriksen-Goldsen et al. (2013) [2]	USA	Health disparities study/population-based analysis	Lesbian, gay, and bisexual older adults	LGB adults aged 50 years and older; data from the 2013 and 2014 National Health Interview Survey	Health disparities among LGB older adults, including higher rates of disability and mental distress	8	High
Stinchcombe et al. (2017) [28]	Canada	Scoping review	24 studies	Palliative care and grief in LGBT older adults	Highlights bereavement-related needs in older adults and the relevance of palliative care contexts.	9	High
Wheat & Thacker (2019) [38]	USA	Counseling/conceptual practice.	No empirical sample; clinical vignette and conceptual discussion.	LGBTQ+ losses and meaning reconstruction.	Proposes meaning reconstruction as a therapeutic resource for LGBTQ+ losses.	9	High
Alasuutari (2021) [24]	Finland	Interpretative qualitative	10 interviews	Queer partner loss and post-mortem intimacy	Extends understanding of continuing bonds and queer post-mortem intimacy after bereavement.	9	High
Valenti et al. (2021) [39]	USA	Qualitative	21 older LGB women	LGB widowhood in later life	Shows disenfranchised grief and the importance of affirming support in older lesbian and bisexual widows.	8	High
Siconolfi et al. (2024) [10]	USA	Quantitative	Sexual minority men aged 40+ (N = 1071)	End-of-life planning, not bereavement-specific	Shows how relationship status, legal protection, and primary care access shape planning	9	High
Pentaris & Patlamazoglou, (2023) [25]	Australia	Qualitative; interpretative phenomenological.	10 participants: 6 gay men and 4 lesbians, aged 56 to 82 years.	Bereavement in later life after the death of a same-gender partner.	Explores symbolic losses and unsolicited gains after partner death.	7	Moderate
Lutz & Ehrlich (2023) [12]	USA	Narrative review	Not reported	LGBTQIA+ patient discrimination and education	Emphasizes provider education and reduction of discrimination in care settings.	9	High
Bristowe et al. (2023) [22]	UK	Descriptive qualitative	21 participants	Loss of spouse or civil partner among LGBT people	Highlights lack of recognition and unwanted outing during bereavement.	9	High
Valenti et al. (2023) [23]	USA	Descriptive qualitative	16 LGB widows	Older lesbian and bisexual women	Contrasts the role of chosen families with that of biological families in bereavement support.	8	High
Timmins et al. (2023) [27]	UK	Cross-sectional study	*n* = 562 bereaved civil partners/spouses	Psychological distress after partner bereavement	Shows greater psychological distress among same-gender bereaved partners.	8	High
Rosa et al. (2023) [11]	USA	Systematic mixed-methods review	13 studies retained	Serious illness and identity-safe disclosure	Supports chosen-family inclusion and identity-safe disclosure in care.	7	Moderate
Caballero Guzmán and Restrepo Duque (2023) [31]	Colombia	Review	LGBTQ+ population	Palliative care and end-of-life care	Highlights palliative care needs, access barriers, and the need for inclusive, person-centred care	9	High
De Jong et al. (2024) [40]	Canada	Scoping review	31 studies	Palliative care in 2SLGBTQIA+ individuals	Synthesizes discrimination, disenfranchised grief, and provider-training gaps.	8	High
Stahl et al. (2026) [26]	USA	Case narrative	1 case	Older gay man, grief, and digital tools	Illustrates social isolation and the potential role of digital tools in grief support.	6	Low

## Data Availability

No new datasets were created or analyzed in this study. All information included in this scoping review comes from previously published sources cited in the manuscript.

## Supplementary Materials

The following supporting information can be downloaded at: https://www.mdpi.com/article/10.3390/healthcare14121758/s1, File S1: The completed PRISMA-ScR checklist [5].

## Abbreviations

The following abbreviations are used in this manuscript:

LGBTIQ+	lesbian, gay, bisexual, transgender, intersex, queer and other identities
PRISMA-ScR	Preferred Reporting Items for Systematic Reviews and Meta-Analyses extension for Scoping Reviews
JBI	Joanna Briggs Institute
PCC	Population–Concept–Context
