# Supplementary material for: Bereavement Following the Loss of a Partner Among LGBTIQ+ Individuals: A Scoping Review of the Evidence (2016–2026)
Source: Healthcare (Basel). 2026 Jun 18;14(12):1758. doi: 10.3390/healthcare14121758 (PMC13299162; doi:10.3390/healthcare14121758)
Supplement: Supplementary file 1 [file healthcare-14-01758-s001.zip › healthcare-4353410-supplementary.pdf]

PRISMA-ScR Checklist (Tricco et al., 2018)

TITLE

Item	Description	Location in Manuscript
1	Identify the report as a scoping review.	1

ABSTRACT

Item	Description	Location in Manuscript
2	Structured summary including background, objectives, eligibility, sources, methods, results, conclusions.	1

INTRODUCTION

Item	Description	Location in Manuscript
3	Describe rationale in context of existing knowledge.	2
4	State objectives using PCC (Population, Concept, Context).	3

METHODS

Item	Description	Location in Manuscript
5	Protocol and registration.	5
6	Eligibility criteria.	3
7	Information sources.	4
8	Search strategy.	4

9	Selection process.	5
10	Data charting process.	6
11	Data items.	6
12	Critical appraisal (optional).	N/A
13	Synthesis of results.	8

RESULTS

Item	Description	Location in Manuscript
14	Selection of sources of evidence.	6
15	Characteristics of sources.	6
16	Critical appraisal (if applicable).	N/A
17	Results of individual sources.	8
18	Synthesis of results.	8

DISCUSSION

Item	Description	Location in Manuscript
19	Summary of evidence.	9
20	Limitations.	9
21	Conclusions.	10

FUNDING

Item	Description	Location in Manuscript
22	Funding sources.	11
